# Mobilization of monocytic myeloid-derived suppressor cells is regulated by PTH1R activation in bone marrow stromal cells

**DOI:** 10.1038/s41413-023-00255-y

**Published:** 2023-04-21

**Authors:** Eun Jung Lee, Kyoung Jin Lee, Seungpil Jung, Kyong Hwa Park, Serk In Park

**Affiliations:** 1 Department of Biochemistry and Molecular Biology, Korea University College of Medicine, Seoul, Republic of Korea; 2 The BK21 Graduate Program, Department of Biomedical Sciences, Korea University College of Medicine, Seoul, Republic of Korea; 3 Division of Breast and Endocrine Surgery, Department of Surgery, Korea University Anam Hospital, Seoul, Republic of Korea; 4 Division of Oncology and Hematology, Department of Internal Medicine, Korea University Anam Hospital, Seoul, Republic of Korea; 5 Vanderbilt Center for Bone Biology, Vanderbilt University School of Medicine, Nashville, TN USA

**Keywords:** Pathogenesis, Bone cancer

## Abstract

Myeloid-derived suppressor cells (MDSCs) are bone marrow (BM)-derived immunosuppressive cells in the tumor microenvironment, but the mechanism of MDSC mobilization from the BM remains unclear. We investigated how BM stromal cell activation by PTH1R contributes to MDSC mobilization. PTH1R activation by parathyroid hormone (PTH) or PTH-related peptide (PTHrP), a tumor-derived counterpart, mobilized monocytic (M-) MDSCs from murine BM without increasing immunosuppressive activity. In vitro cell-binding assays demonstrated that α4β1 integrin and vascular cell adhesion molecule (VCAM)-1, expressed on M-MDSCs and osteoblasts, respectively, are key to M-MDSC binding to osteoblasts. Upon PTH1R activation, osteoblasts express VEGF-A and IL6, leading to Src family kinase phosphorylation in M-MDSCs. Src inhibitors suppressed PTHrP-induced MDSC mobilization, and Src activation in M-MDSCs upregulated two proteases, ADAM-17 and MMP7, leading to VCAM1 shedding and subsequent disruption of M-MDSC tethering to osteoblasts. Collectively, our data provide the molecular mechanism of M-MDSC mobilization in the bones of tumor hosts.

## Introduction

Antitumoral T-cell immunity in the tumor microenvironment is counteracted by several types of immunosuppressive bone marrow-derived cells, such as tumor-associated macrophages, tumor-associated neutrophils and myeloid-derived suppressor cells (MDSCs).^1–3^ Although these cells have important functions in tumor tissue, key questions remain unanswered, such as how bone marrow-derived immune cells are regulated and/or mobilized *within* the bone marrow of tumor hosts. The aim of this study was to elucidate the molecular mechanism of MDSC mobilization within the bone marrow of breast cancer patients and mouse models.

MDSCs are a subset of immature myeloid-lineage bone marrow cells in tumor tissue and are known for their T-cell suppressive activity by expressing arginase 1 (Arg1), transforming growth factor (TGF) β, inducible nitric oxide synthase (iNOS), reactive oxygen species (ROS), etc.^4^ Numerous lines of evidence clearly support the existence and function of MDSCs in human cancer patients and murine tumor models.^5–7^ Two major subpopulations of MDSCs, polymorphonuclear (PMN-, or granulocytic, G-) and monocytic (M-) types, are currently accepted. G-MDSCs are more prevalent and suppress T-cell responses in an antigen-specific manner, whereas M-MDSCs are more suppressive on a per cell basis and in both antigen-specific and antigen-nonspecific manners. Notwithstanding recent exponentially increasing publications on MDSCs, the generation and development of MDSCs in cancer patients remain a crucial topic of further investigation.^8,9^ The current hypothesis of MDSC development is a two-stage model based on two distinct yet interconnected groups of signals, e.g., expansion of immature myeloid cells in the bone marrow and subsequent activation/acquisition of suppressive activity.^10^ Several chemokines and cytokines, particularly those regulating myelopoiesis and differentiation of myeloid cells, have been shown to expand MDSCs. For example, multiple groups independently reported that granulocyte-macrophage colony-stimulating factor (GM-CSF), granulocyte CSF (G-CSF) and/or interleukin (IL) 6 increase MDSC accumulation in tumor tissue.^11–13^ In addition, tumor-derived factors, including C-C chemokine ligand (CCL) 2, transforming growth factor (TGF)-β, IL-4, etc., have been demonstrated to increase MDSCs.^8,14^ Stomach-specific IL-1β overexpression increased MDSC accumulation during gastric cancer initiation and progression.^15^ However, very little is known about how cytokines/chemokines trigger molecular pathways leading to MDSC mobilization within the bone marrow. The majority of previous studies on the mechanism of MDSC mobilization focused on the intrinsic effects of MDSCs, i.e., via receptors expressed on the cell surface of MDSCs. Svoronos et al. demonstrated that estrogen receptor α expressed by human and murine bone marrow myeloid precursors activates the signal transducer and activation of transcription (STAT) 3 pathway via Janus kinase (JAK) 2 and Src nonreceptor tyrosine kinase.^16^ Activation of the cannabinoid receptors CB1 and CB2 expressed on immune cells mobilized functional MDSCs, contributing to the immunomodulatory effects of cannabinoids.^17^ Stimulator of interferon genes (STING) and the type I interferon pathway induce M-MDSC accumulation in tumor tissue via C-C chemokine receptor (CCR) 2.^18^

Given that MDSCs originate in the bone marrow of tumor hosts, stromal cells comprising the bone microenvironment are thought to participate in the initial phase of MDSC development. We have previously demonstrated that prostate tumor-derived parathyroid hormone-related peptide (PTHrP) correlates with MDSC accumulation in prostate tumor tissues, a process that involves activation of the PTHrP receptor, PTH1R, in osteoblasts.^19^ Briefly, prostate tumor-derived PTHrP activates osteoblasts, the main cell type expressing PTH1R in the bone microenvironment, leading to the expression of vascular endothelial growth factor (VEGF)-A and IL6. Consequently, MDSCs gain increased angiogenic potential to contribute to prostate tumor growth and angiogenesis. In addition to the functional activation of MDSCs, we observed that PTHrP rapidly increases MDSCs in the circulation of tumor hosts, suggesting that osteoblastic PTH1R activation potentially contributes to the expansion and/or mobilization of MDSCs. In this manuscript, we further delved into the molecular mechanism of PTH1R-dependent MDSC mobilization in tumor hosts using in vitro cell binding assays, in vivo models and human breast cancer patient-derived MDSCs.

## Results

### PTHrP increased the number of M-, but not G-, MDSCs in the circulation

**Fig. 1:**
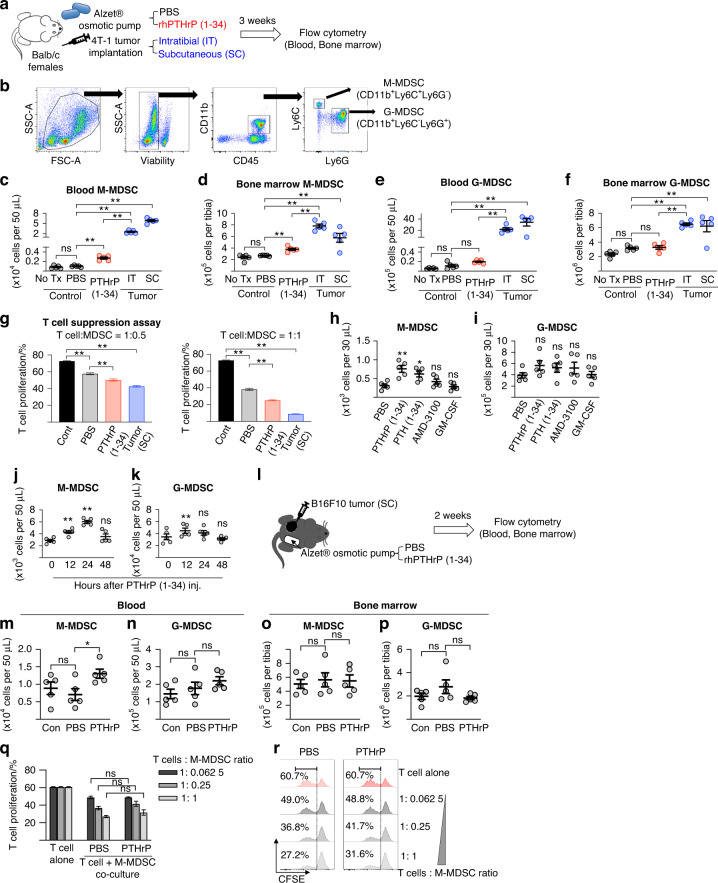
PTHrP increases the number of monocytic, but not granulocytic, MDSCs in the circulation and bone marrow, and PTHrP does not further augment the immunosuppressive activity of monocytic MDSCs in tumor-bearing mice. **a** Schematic representation of the in vivo experiments shown in Panels **c**–**g**. **b** Flow cytometric analysis gating scheme. CD11b^+^Ly6C^+^Ly6G^−^ or CD11b^+^Ly6C^−^Ly6G^+^ blood or bone marrow cells in the viable CD45^+^ cell population were defined as M- or G-MDSCs, respectively. **c**–**f** Flow cytometric results showing that three weeks of continuous infusion of PTHrP (1–34; 80 μg·kg^−1^ daily) increased the number of M-, but not G-, MDSCs in the blood and bone marrow compared with no treatment or PBS infusion. **g** T-cell suppression assay using CFSE-labeled T cells (isolated from the spleens of nontumor-bearing Balb/C donor mice) stimulated by IL-2 (10 IU per mL) and Dynabeads® CD3/CD28 T-cell activators. **h**, **i** A single administration of rhPTHrP (1–34; 80 μg·kg^−1^) or rhPTH (1-34; 80 μg·kg^−1^) increased the number of M-, but not G-, MDSCs in the blood within 24 h compared with the PBS control. **j**, **k** Time course of the M- or G-MDSC increase in the circulation after a single administration of PTHrP (1–34; 80 µg·kg^−1^). **l** Schematic representation of the experiment shown in Panels (**m**–**r**). **m**–**p** PTHrP infusion increased M-, but not G-, MDSCs in the blood circulation but not in the bone marrow compared with those of the tumor alone (control) or PBS-infused groups. **q**, **r** T-cell proliferation assay using CFSE-labeled T cells (isolated from the spleens of nontumor-bearing C57BL6 donor mice) cocultured with M-MDSCs isolated from B16F10 tumor-bearing mice treated with PBS or PTHrP in the presence of IL-2 (10 IU per mL) and Dynabeads® CD3/CD28 T-cell activators. T-cell proliferation was measured by flow cytometry and presented in a bar graph (**q**) and representative histograms (**r**). All experiments used 7-week-old female Balb/c (Panels **a**‒**k**) or male C57BL6 (Panels **l**–**r**) mice (*n* = 5 per group). Dots represent individual samples, and horizontal lines represent the mean ± SEM. Bar graphs (**g**, **q**) are the mean ± SEM (*n* = 3 per group). *P < 0.05; **P < 0.01; ns nonsignificant; Mann‒Whitney *U* test

To investigate the specific effects of PTHrP and to rule out many other tumor-derived factors that potentially affect MDSCs, we first infused recombinant PTHrP (amino acids 1-34, a PTH1R receptor-binding fragment) or control PBS diluent continuously for three weeks using in vivo subcutaneous implantable pumps in tumor-naïve mice (Fig. 1a). In parallel, subcutaneous or intratibial 4T1 tumor-bearing mice were used as a positive controls for MDSC induction (Fig. 1b–f). Flow cytometric analyses show that PTHrP administration significantly increased the number of M-MDSCs, but not G-MDSCs, in both blood circulation and the femoral bone marrow. The increase was statistically significant compared with the no-treatment or PBS-treated control groups but was less prominent compared with murine subcutaneous or intratibial 4T1 tumor-bearing mice, suggesting that PTHrP is not the only but one of the M-MDSC mobilizing factors. Because MDSCs are functionally defined as T-cell-suppressive immature myeloid cells, we performed a T-cell suppression assay to confirm that MDSCs isolated from PTHrP-infused mice were functional. Fig. 1g shows that M-MDSCs isolated from the PTHrP-treated mice suppressed in vitro T-cell proliferation compared with MDSCs isolated from the PBS-treated control mice. The T-cell suppressive activity was less prominent than that of MDSCs isolated from the subcutaneous 4T1 tumor-bearing mice. We subsequently tested whether PTH (amino acids 1–34, a receptor-binding fragment), whose receptor, PTH1R, is shared with PTHrP, also increases M- and G-MDSCs in vivo. A single subcutaneous administration of both PTH(1-34) or PTHrP(1-34) significantly increased M-, but not G-, MDSCs in the circulation of mice within 24 h. In contrast, AMD3100 (plerixafor, a CXCR4 inhibitor) and granulocyte-macrophage colony stimulating factor (GM-CSF), two clinically used hematopoietic stem cell mobilizing agents, did not increase M- or G-MDSCs, indicating that M-MDSC mobilization is specific to PTH1R activation (Fig. 1h, i). We also examined whether PTH or PTHrP increases other types of hematopoietic lineage cells. Neither PTH(1-34) nor PTHrP(1-34) affected granulocytes, lymphocytes (B cells and CD3, 4, 8 or NK T cells) or white blood cell (WBC) differential counting (Supplemental Fig. 2) in mice, indicating that PTH1R activation specifically increases M-MDSCs. Figure 1j, k shows that the PTHrP(1-34)-induced M-MDSC increase in the circulation occurred rapidly within 12 h after injection and reached a nadir at the 48-hour time point, suggesting that the rapid increase is potentially attributable to mobilization, not proliferation, of M-MDSCs.

### PTHrP did not increase the immunosuppressive activity of M-MDSCs in tumor-bearing mice

MDSCs exist only in pathologic conditions such as inflammation and cancer, but the experiments in Fig. 1a–k were performed in tumor-naïve mice, and M-MDSCs isolated from PTHrP-infused tumor-naïve mice were immunosuppressive. We thus re-examined the effects of PTHrP in tumor-bearing mice. We chose B16F10 murine melanoma cells because of undetectable endogenous PTHrP expression. B16F10 tumor-bearing mice were continuously infused with recombinant PTHrP(1-34) or PBS for two weeks, followed by flow cytometric analysis (Fig. 1l–p). The PTHrP-treated mice had significantly increased numbers of M- but not G-MDSCs in the circulation. However, contrary to the data from tumor-naïve mice shown in Fig. 1d, PTHrP did not increase M-MDSCs in the bone marrow. We reasoned that MDSCs in the bone marrow had already maximally expanded in response to the 2-week subcutaneous tumor burden, and the effects of exogenous PTHrP addition did not generate further noticeable changes (Fig. 1o).

We next tested whether the T-cell-suppressive activity of MDSCs, a hallmark of functional MDSCs, was affected by PTHrP treatment. In Fig. 1q–r, in the in vitro T-cell suppression assay with MDSCs isolated from two-week PTHrP- or PBS-infused mice carrying B16-F10 tumors, MDSCs suppressed T-cell proliferation, while PTHrP treatment did not augment T-cell suppressive function in tumor-bearing mice. These findings may seem to contradict the data in Fig. 1g showing that PTHrP treatment increased the immunosuppressive activity of M-MDSCs in tumor-naïve mice, but we reasoned that the effects of PTHrP on the immunosuppressive activity of MDSCs may not be prominent in tumor-bearing mice because PTHrP may not be the only tumor-derived factor for MDSC function, and cytokines and growth factors released by tumor cells may increase and mask the effects of PTHrP. In contrast, PTHrP-dependent M-MDSC mobilization was evident in both tumor-naïve and tumor-bearing mice, supporting the validity of our work. Collectively, we conclude that PTHrP mobilizes M-MDSCs and increases the immunosuppressive activity of M-MDSCs, while the effect on immunosuppressive activity is not prominent in tumor-bearing mice. We conclude that PTHrP(1-34) induced MDSC mobilization without enhancing the immunosuppressive functions of M-MDSCs.

### Activation of osteoblasts via PTH1R ligands released M-MDSCs binding to osteoblasts in vitro

**Fig. 2:**
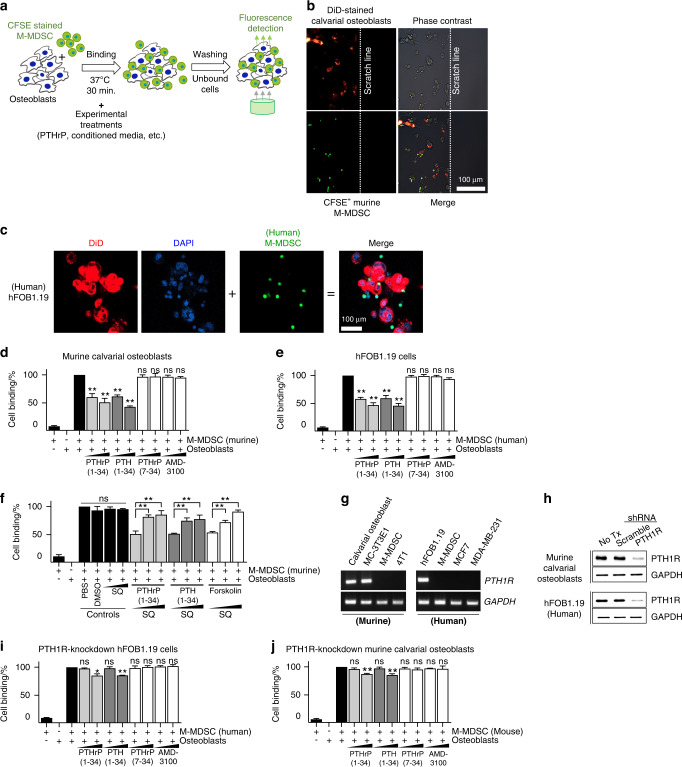
Activation of osteoblasts via PTH1R ligands (PTH or PTHrP) releases M-MDSCs binding to osteoblasts. **a** Schematic representation of the in vitro cell binding assay. Murine or human M-MDSCs were isolated by flow cytometric sorting from 4T1 tumor-bearing mouse tibia or breast cancer patient blood, followed by CFSE labeling and coculture with primary calvarial (murine) or hFOB1.19 (human) osteoblast monolayer cultures, respectively. **b**, **c** Microscopic images of murine primary calvarial osteoblasts (**b**) or human hFOB1.19 (**c**) osteoblasts (Vibrant® DiD-NIR dye; orange) cocultured with murine or human M-MDSCs (CFSE; green), respectively, for 15 min. **d**, **e** Addition of rhPTHrP(1-34) or rhPTH(1-34), both functional ligands for the common receptor PTH1R, released M-MDSC binding to osteoblasts. **f** Effects of rhPTHrP (1-34, 1 or 10 nmol·L^−1^) or rhPTH (1-34, 1 or 10 nmol·L^−1^) on M-MDSC and osteoblast binding release were inhibited by SQ22,356 (SQ, 0, 10 or 100 μmol·L^−1^), an inhibitor of adenylate cyclase downstream of PTH1R. **g** Semiquantitative RT‒PCR showing *PTH1R* expression in murine (primary calvarial or MC3T3E1 cell line) and human hFOB1.19 osteoblasts but not in M-MDSCs or breast tumor cells (4T1 murine or MCF7 and MDA-MB-231 human cell lines). **h**–**j** PTH1R knockdown blunted the effects of PTHrP (1-34, 1 or 10 nmol·L^−1^) or PTH (1-34, 1 or 10 nmol·L^−1^) on MDSC-osteoblast binding release. **h** Western blotting analysis showing decreased expression of PTH1R in murine or human osteoblasts transduced with PTH1R-specific shRNA expression lentiviral vectors. Untreated or scrambled sequence expression vector-transfected cells were used as controls. **i**, **j** In vitro cell binding assay using *PTH1R* knockdown osteoblasts. Data are the mean ± SEM (*n* = 3 per group). **P < 0.01; ns nonsignificant. One-way ANOVA with multiple group comparisons

The data in Fig. 1 collectively demonstrate that PTH1R activation by PTHrP or PTH specifically increases M-MDSCs in the circulation. In addition, the kinetics of the PTHrP-induced increase in MDSCs (Fig. 1h–k) suggest that the rapid increase in M-MDSCs (i.e., within 12 h after PTHrP injection) is mediated by mobilization of M-MDSCs from the bone marrow into circulation rather than by proliferation and/or expansion of M-MDSCs in the bone marrow. Given that PTH1R is predominantly expressed by osteoblasts in bone, we subsequently hypothesized that interactions between osteoblasts and M-MDSCs are disrupted by PTH1R activation, leading to disengagement of M-MDSCs from the bone marrow into the circulation. To test this hypothesis, we reconstituted osteoblast-MDSC interactions in vitro and tested the effects of PTH or PTHrP (Fig. 2a and Fig. S3a). Microscopic images in Fig. 2b, c show that human or murine M-MDSCs bound to the cultured osteoblast monolayer. The addition of PTHrP(1-34) or PTH(1-34) reduced the binding between M-MDSCs and osteoblasts in a dose-dependent manner, while PTHrP(7-34, a nonreceptor binding fragment) or AMD-3100 did not reduce the binding. Moreover, the effects of PTHrP(1-34) or PTH(1-34) on M-MDSC and osteoblast binding release were inhibited by SQ22356, an inhibitor of adenylate cyclase downstream of PTH1R. Conversely, forskolin, an adenylate cyclase activator, released M-MDSCs and osteoblast binding, which was blocked by the addition of SQ22356 (Fig. 2d–f, Fig. S3b and c and Movie S1). We further confirmed whether activation of PTH1R, a common receptor for PTH and PTHrP, is essential for the binding release of M-MDSCs from osteoblasts. Indeed, *PTH1R* knockdown in osteoblasts significantly blunted the effects of PTHrP(1-34) or PTH(1-34) on MDSC-osteoblast binding release (Fig. 2g–j). Overall, the data in Fig. 2 support that PTH1R is critical to the effects of PTH or PHTrP on M-MDSC mobilization.

### Electron microscopy demonstrates physical binding between M-MDSCs and osteoblasts via cell adhesion

**Fig. 3:**
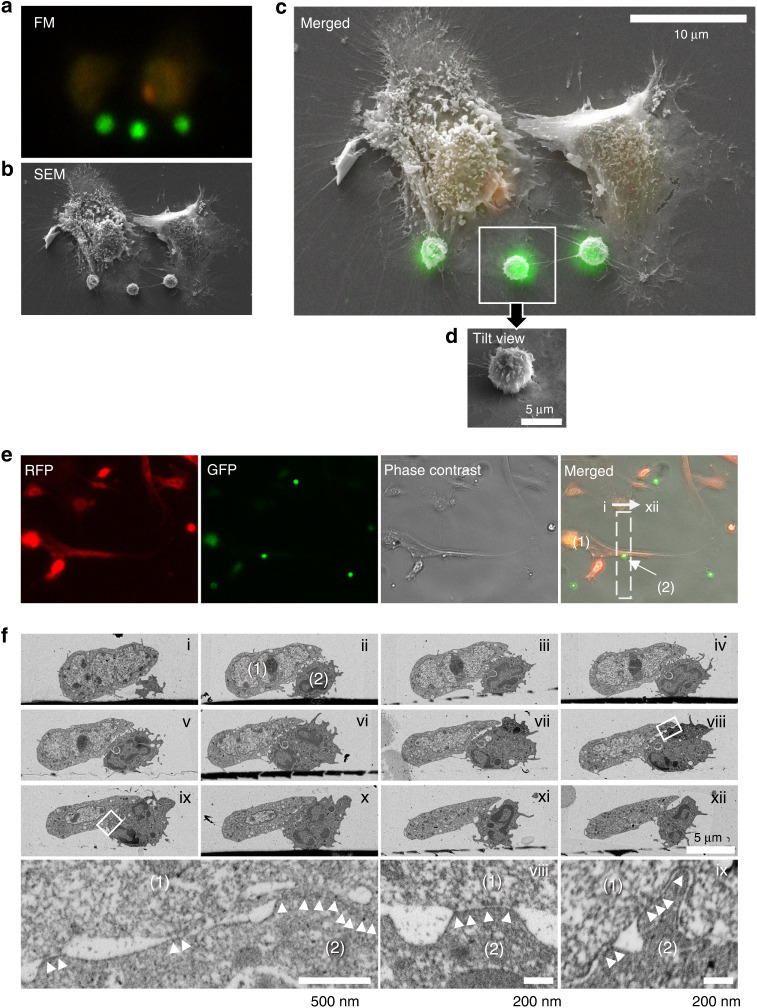
Electron microscopic images demonstrate physical binding between M-MDSCs and osteoblasts. Murine M-MDSCs (CFSE; 1 × 10^5^ cells) were added to adherent murine calvarial osteoblast (Vibrant® DiD-NIR dye; orange; 1 × 10^5^ cells) culture, followed by 30 min of incubation at 37 °C and washing of unbound cells with PBS. **a**–**d** Correlative light and electron microscopy (CLEM) images showing physical binding between osteoblasts (adherent cells) and M-MDSCs (green). Cells were imaged with a widefield fluorescence microscope and subsequently processed and imaged with a scanning electron microscope. Two images (**a**, **b**) were overlaid and are shown in **(c)**. Three M-MDSCs do not seem to physically bind to osteoblasts in fluorescence microscopic images (**a**) but indeed bind to the cell body of osteoblasts (**b**–**d**). Inbox (**d**) shows the tilt view of the cell marked with a white box. FM, fluorescence microscopy. SEM, scanning electron microscopy. (**e**) DiD-labeled osteoblasts and CFSE-labeled M-MDSCs were imaged using RFP and GFP channels, respectively, followed by merging with a phase-contrast microscopic image. One representative osteoblast (indicated by 1) and one representative M-MDSC (indicated by 2, in the dotted line box) were further imaged by backscattered electron (BSE)-SEM serial ultrathin sections (images from i to xii in Panel **f**). **f** Serial ultrathin sections of the area indicated by the dotted line box in (**e**). Enlarged images of the part where two cells were attached. White arrowheads indicate cell adhesion regions. (1), osteoblasts. (2), M-MDSCs

We further confirmed the specificity of M-MDSC and osteoblast binding by electron microscopy. Correlative light and electron microscopy (CLEM) images in Fig. 3a–d show physical binding between osteoblasts (adherent cells) and M-MDSCs (green). Three M-MDSCs that seemingly appear not to bind directly to osteoblasts under a fluorescence microscope (Fig. 3a) indeed bind to the cell body of osteoblasts under an electron microscope (Fig. 3b–d). Furthermore, serial ultrathin sections of M-MDSCs and osteoblasts confirmed that M-MDSC and osteoblast binding occurs through cellular adhesion, not simple colocalization (Fig. 3f). These data support that M-MDSCs bind to osteoblasts via cell adhesion molecules.

### M-MDSCs reside in the endosteal surface of bone marrow via VCAM1 and Integrin β1

Based on the data in Figs. 1–3, we reasoned that M-MDSCs are bound to osteoblasts in the bone marrow of tumor hosts, and the binding becomes disengaged upon PTH1R activation by tumor-derived PTHrP. We subsequently searched for cell-to-cell adhesion molecules associated with M-MDSC and osteoblast binding. Very late antigen (VLA)-4 is a dimer of integrin α4 (CD49D) and β1 (CD29) subunits and is expressed by hematopoietic lineage cells, including hematopoietic stem cells (HSCs) and monocytes. Stromal-derived factor (SDF)1, also known as C-X-C motif chemokine (CXCL)12, activates VLA4, leading to conformational changes and binding to vascular cell adhesion molecule (VCAM)1, the primary ligand of VLA4. The VLA-4 and VCAM1 axis regulates HSC homing, dormancy and mobilization.^20,21^ Recently, the Varner group showed that VLA4 is expressed by MDSCs, playing key roles in recruitment to tumor tissues, angiogenesis, and immunosuppressive activity.^22–25^ We subsequently tested whether the VLA-4 (α4β1 integrin) and VCAM1 axis plays a role in MDSC mobilization from the bone marrow into the circulation.

**Fig. 4:**
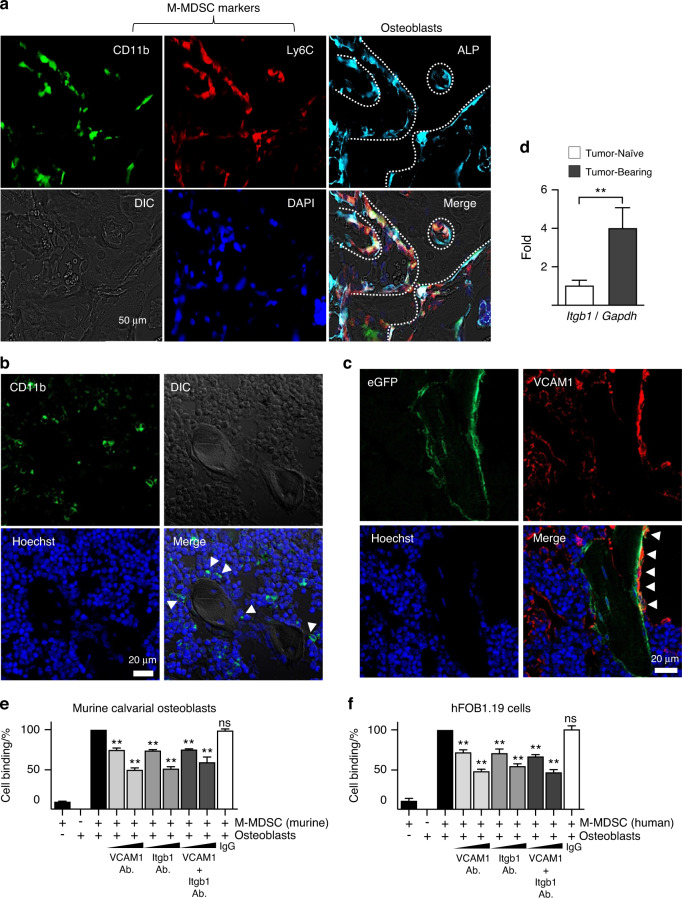
M-MDSCs reside in the endosteal surface of bone marrow via VCAM1 and Integrin β1. **a** Immunofluorescence staining of the tibia of Balb/c female mice with 4T1 breast cancer bone metastasis. White dotted lines indicate the endosteal surface. Anti-mouse CD11b (green), anti-Ly6C (red) and anti-ALP (teal) antibodies were labeled with Alexa Fluor 488, Alexa Fluor 594 and Cy5.5 fluorescence dye, respectively. Images were captured using a fluorescence microscope (EVOS FL Auto 2). **b** Immunofluorescence staining of the tibia of 4T1 tumor-bearing Balb/c mice. CD11b (green)-positive cells were imaged and overlaid with differential interference contrast (DIC) and Hoechst 33342 counterstained images. Some CD11b-positive cells are localized close adjacent to endosteal lining cells (indicated with white triangles). Images were captured using a confocal microscope (Zeiss LSM-900). **c** Immunofluorescence staining of the tibia of Osteocalcin (*Ocn*)-*Cre*:*ROSA*^*mT/mG*^ reporter female mice carrying B16F10 tumors. Osteocalcin (Ocn, green) and VCAM1 (Alex Fluor 647, red) expression colocalized (indicated by white triangles), supporting that endosteal lining osteoblasts express VCAM1. Hoechst 33342 (blue) counterstaining for nuclei. Images were captured using a confocal microscope (Zeiss LSM-900). **d** Expression of β1 integrin mRNA measured by RT‒qPCR in M-MDSCs isolated from the bone marrow of 4T1 tumor-bearing tibia. CD11b^+^Ly6C^+^Ly6G^−^ cells from untreated tumor-naïve mice were used as controls. Data are the mean ± SEM (*n* = 5 per group). **P < 0.01, Student’s *t* test. **e**, **f** In vitro cell binding assay showing that VCAM1 or integrin β1 neutralizing antibodies (1 or 2 μg·mL^−1^) block M-MDSC and murine (**e**) or human (**f**) osteoblast binding. Nonspecific IgG was used as a control. Data are the mean ± SEM (*n* = 5 per group). **P < 0.01, one-way ANOVA with multiple group comparisons

The Fig. 4a immunofluorescence images of the tibia of Balb/c female mice carrying 4T1 breast cancer revealed that alkaline phosphatase (ALP)-positive osteoblasts on the endosteal surface colocalized with CD11b^+^Ly6C^+^ M-MDSCs. In addition, a fraction of CD11b^+^ cells (including MDSCs) located adjacent to the endosteal lining osteoblasts (Fig. 4b) and endosteal lining osteoblasts expressed VCAM1 (Fig. 4c). In our subsequent in vitro cell binding assay, anti-VCAM1 or anti-integrin β1 neutralizing antibodies blocked M-MDSC and osteoblast binding dose-dependently (Fig. 4e, f), supporting that VCAM1 (expressed on osteoblasts) and VLA-4 (integrin α4β1, expressed on M-MDSCs) mediate the binding between osteoblasts and M-MDSCs.

### Binding between M-MDSCs and bone marrow stromal cells is dependent on VCAM1 expression, but PTH1R ligand (PTH or PTHrP)-induced release is dependent on PTH1R expression

**Fig. 5:**
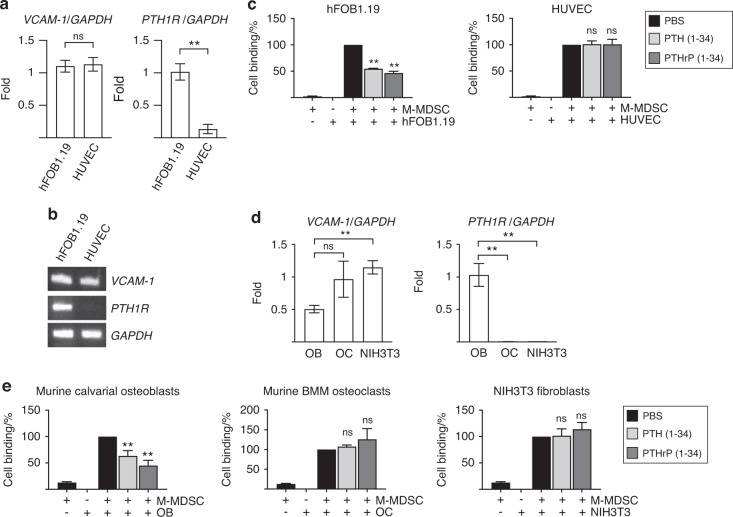
Binding between M-MDSCs and bone marrow stromal cells is dependent on VCAM1 expression, but PTH1R ligand (PTH or PTHrP)-induced release is dependent on PTH1R expression. **a**, **b** Expression of VCAM1 and PTH1R in hFOB1.19 (human osteoblasts) and HUVECs (human umbilical vein endothelial cells) analyzed by quantitative (**a**) and semiquantitative (**b**) RT‒PCR. Both hFOB1.19 cells and HUVECs expressed VCAM1, but only hFOB1.19 cells expressed PTH1R. Data are the mean ± SEM (*n* = 3 per group). **P < 0.01, one-way ANOVA with multiple group comparisons. NS, nonsignificant. **c** In vitro cell binding assay showing that rhPTH(1-34) or rhPTHrP(1-34) released M-MDSC binding to osteoblasts in hFOB1.19 cells, but not in HUVECs, compared with the PBS control. M-MDSCs were isolated from 4T1 tumor-bearing murine tibiae by flow cytometric sorting. **d** Expression of VCAM1 and PTH1R in murine calvarial osteoblasts (OB), murine bone marrow monocyte (BMM)-derived osteoclasts (OC) and NIH3T3 murine fibroblasts analyzed by quantitative RT‒PCR. OB, OC and NIH3T3 cells expressed VCAM1, but only OB cells expressed PTH1R. Data are the mean ± SEM (*n* = 3 per group). **P < 0.01, Student’s *t* test. NS nonsignificant. **e** In vitro cell binding assay showing that rhPTH (1-34, 10 nmol·L^−1^) or rhPTHrP (1-34, 10 nmol·L^−1^) released M-MDSC binding to osteoblasts in OBs but not in OCs or NIH3T3 cells compared with the PBS control. M-MDSCs were isolated from 4T1 tumor-bearing murine tibiae by flow cytometric sorting. Data are the mean ± SEM (*n* = 3 per group). *P < 0.05, **P < 0.01, one-way ANOVA with multiple group comparisons. NS nonsignificant

Notwithstanding the in vivo histological data in Fig. 4, VCAM-1 expression is not confined to the endosteal lining osteoblasts, and not all M-MDSCs in the bone marrow bind to osteoblasts in vivo or in human patients. We subsequently examined whether M-MDSCs potentially bind to other cell types expressing VCAM1 (e.g., osteoclasts and fibroblasts). Figure 5a, b, d demonstrates that cells in the bone marrow, such as endothelial cells, osteoclasts, and fibroblasts, express VCAM-1, but only osteoblasts express PTH1R. In vitro cell binding assays (Fig. 5c, e) showed that only PTH/PTHrP stimulation released M-MDSC binding from osteoblasts, supporting that MDSC-bone marrow stromal cell binding release is dependent on PTH1R activation. Collectively, these data indicate that a fraction of M-MDSCs bound to the endosteal surface in the bone marrow are mobilized by PTH1R activation in osteoblasts, which potentially is not the only mechanism for M-MDSC mobilization in cancer patients. Thus, the anti- and protumoral immunity balance is likely not regulated by one gold standard mechanism; instead, the balance is regulated by multiple overlapping yet compensatory mechanisms.

### Ectopic expression of VCAM1 confers M-MDSC binding capability

**Fig. 6:**
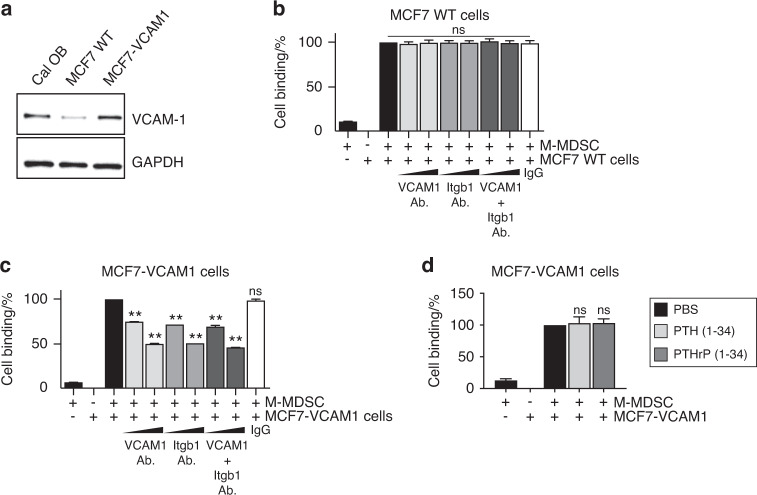
Ectopic expression of VCAM1 confers M-MDSC binding capability. **a** Western blotting showing VCAM1 ectopic overexpression in MCF7 breast cancer cells. **b**, **c** In vitro cell binding assay showing that anti-VCAM1 and/or anti-integrin β1 antibodies (1 μg or 2 μg) block M-MDSC binding to VCAM1-overexpressing (**c**) but not parental wild-type (**b**) MCF7 cells. Data are the mean ± SEM (*n* = 3 per group). **P < 0.01, one-way ANOVA with multiple group comparisons. NS nonsignificant. **d** In vitro cell binding assay showing that ectopic expression of VCAM1 in MCF-7 cells confers M-MDSC binding capacity, and the binding is reversed by rhPTH (1-34, 10 nmol·L^−1^) or rhPTHrP (1-34, 10 nmol·L^−1^). Data are the mean ± SEM (*n* = 3 per group). One-way ANOVA with multiple group comparisons. NS nonsignificant

We further confirmed the role of VCAM1 and integrin β1 in osteoblast-M-MDSC binding. MCF7 breast cancer cells that do not express VCAM1 were transfected with a VCAM1 overexpression vector, and ectopic expression was confirmed by Western blotting and immunofluorescence imaging (Fig. 6a). Supplemental Fig. 4a, b shows that murine M-MDSCs bind to VCAM1-overexpressing but not to parental MCF7 cells. In addition, an in vitro cell binding assay showed that anti-VCAM1 and/or anti-integrin β1 antibodies blocked M-MDSC binding to VCAM1-overexpressing but not parental MCF7 cells (Fig. 6b, c). In contrast, an in vitro cell binding assay (Fig. 6d) showed that ectopic expression of VCAM1 in MCF-7 cells confers M-MDSC binding capacity, but the binding is not reversed by rhPTH(1-34) or rhPTHrP(1-34), supporting that M-MDSC binding to bone marrow stromal cells is dependent on VCAM1 and integrin β1 expression but that the binding release is dependent on PTH1R expression.

### Src family kinase activation in M-MDSCs is required for releasing M-MDSCs from osteoblasts

**Fig. 7:**
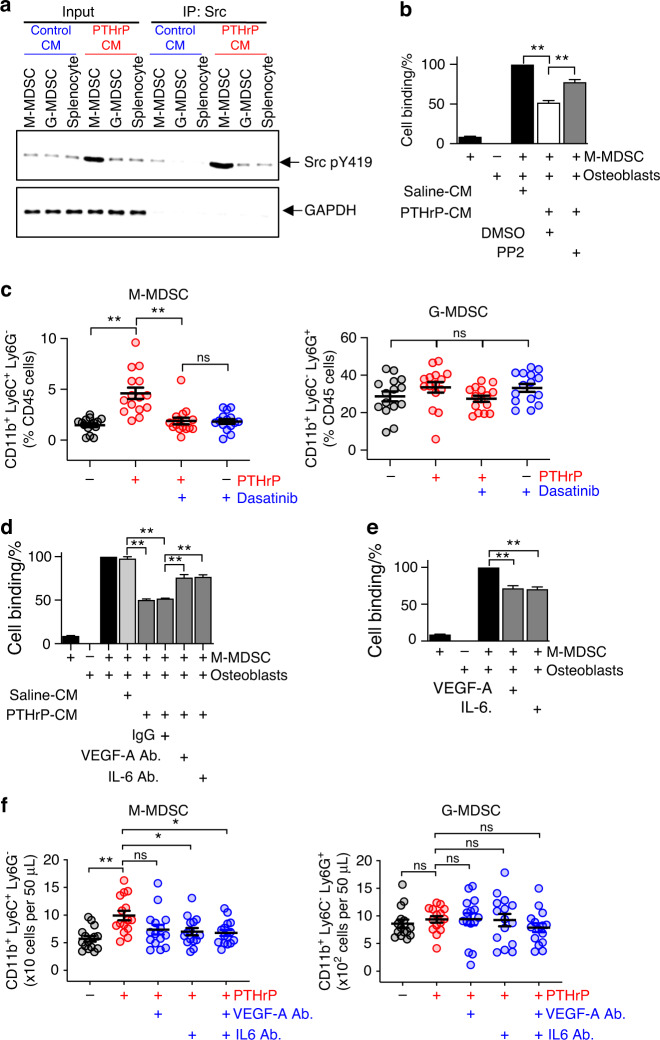
Src family kinase activation via VEGF-A and IL6 in M-MDSCs is required for releasing M-MDSCs from osteoblasts. **a** PTHrP-conditioned media from osteoblasts induced activating phosphorylation of Src family kinase (SFK) at the Y419 residue in M-MDSCs but not in G-MDSCs or splenocytes. **b** In vitro cell binding assay showing that an SFK-specific inhibitor, PP2, reversed the effects of PTHrP-conditioned media on M-MDSC binding release from osteoblasts. CFSE-stained M-MDSCs were cocultured with osteoblasts for 2 h before washing away the unbound cells and measuring the fluorescence intensity. DMSO was used as a vehicle control. Data are the mean ± SEM (*n* = 3 per group). **P < 0.01, one-way ANOVA with multiple group comparisons. **c** Dasatinib, an SFK tyrosine kinase inhibitor, suppressed PTHrP(1-34)-induced M-MDSC mobilization in vivo. Female Balb/C mice were injected with rhPTHrP (1-34; 80 µg·kg^−1^) ± dasatinib (15 mg·kg^−1^, P.O.), followed by flow cytometric quantification of M- and G-MDSCs in blood. Data are from a total of three independent in vivo experiments (*n* = 5 per group × 3 experiments). Dots represent individual samples, and horizontal lines represent the mean ± SEM. **P < 0.01; ns nonsignificant; Mann‒Whitney *U* test. **d** The effects of PTHrP-conditioned media on MDSC-osteoblast binding were reversed by the addition of anti-VEGF-A (2 μg·mL^−1^) or anti-IL6 (2 μg·mL^−1^) antibodies, two osteoblastic cytokines inducing SFK phosphorylation in MDSCs. CFSE-stained M-MDSCs were cocultured with osteoblasts for 2 h before washing away the unbound cells and measuring the fluorescence intensity. Data are the mean ± SEM (*n* = 3 per group). **P < 0.01, one-way ANOVA with multiple group comparisons. **e** Addition of recombinant VEGF-A (2 ng·mL^−1^) and/or IL6 (2 ng·mL^−1^) inhibited MDSC-osteoblast binding in vitro. Data are the mean ± SEM (*n* = 3 per group). **P < 0.01, one-way ANOVA with multiple group comparisons. **f** Anti-VEGF-A or anti-IL6 antibodies suppressed PTHrP(1-34)-induced M-MDSC mobilization in vivo. Female Balb/C mice were injected with rhPTHrP (1-34; 80 µg·kg^−1^) ± anti-VEGF-A (5 mg·kg^−1^, I.P.) and/or anti-IL6 (10 mg·kg^−1^, I.P.) antibodies, followed by flow cytometric quantification of M- and G-MDSCs in blood. Dots represent individual samples, and horizontal lines represent the mean ± SEM (*n* = 15 per group). *P < 0.05; **P < 0.01; ns nonsignificant; Mann‒Whitney *U* test

We have previously demonstrated that prostate cancer-derived PTHrP induces activating phosphorylation of Src family kinase (SFK) at tyrosine residue 419 (Y419) in MDSCs via vascular endothelial growth factor (VEGF)-A and interleukin (IL)-6 expressed by osteoblasts, leading to increased proangiogenic and protumorigenic functions of MDSCs.^19^ We subsequently tested whether SFK activation also plays a role in M-MDSC mobilization. First, we examined whether PTHrP-activated osteoblasts induce pY419 of SFK in M-MDSCs. Murine splenocytes and femoral bone marrow-derived M- and G-MDSCs were isolated from 4T1 tumor-bearing mice by flow cytometry, followed by treatment with 24-hour PTHrP(1-34)- or PBS control-conditioned media from murine calvarial osteoblast cultures. Fig. 7a shows that SFK specifically in M-MDSCs, but not in G-MDSCs or splenocytes, was activated by PTHrP-conditioned media from osteoblasts. In addition, PTHrP-conditioned media decreased the binding between osteoblasts and M-MDSCs in vitro, which was effectively reversed by PP2, an SFK-specific pharmacologic inhibitor (Fig. 7b). In our subsequent in vivo experiment using dasatinib, a tyrosine kinase inhibitor for SFK in clinical use, suppression of SFK effectively inhibited PTHrP(1-34)-induced M-MDSC mobilization in mice (Fig. 7c), suggesting that activating phosphorylation of SFK in M-MDSCs is essential for disengagement from osteoblasts. Furthermore, the effects of PTHrP-conditioned media on disrupting osteoblast-M-MDSC binding were inhibited by the addition of anti-VEGF-A or anti-IL6 neutralizing antibodies (Fig. 7d, e). Indeed, in our subsequent in vivo experiment (Fig. 7f), anti-VEGF-A and/or anti-IL6 antibodies suppressed PTHrP(1-34)-induced M-MDSC mobilization in vivo, supporting that PTHrP-induced VEGF-A and IL6 contribute to M-MDSC mobilization. However, we did not observe statistical significance in the VEGF-A alone treatment group due to two outliers. These data support that SFK activation in M-MDSCs by PTHrP-induced osteoblastic cytokines such as VEGF-A and IL6 is important in M-MDSC mobilization.

### PTHrP-induced M-MDSC mobilization is dependent on a disintegrin and metalloproteinase (ADAM) 17 and matrix metalloprotease (MMP) 7

**Fig. 8:**
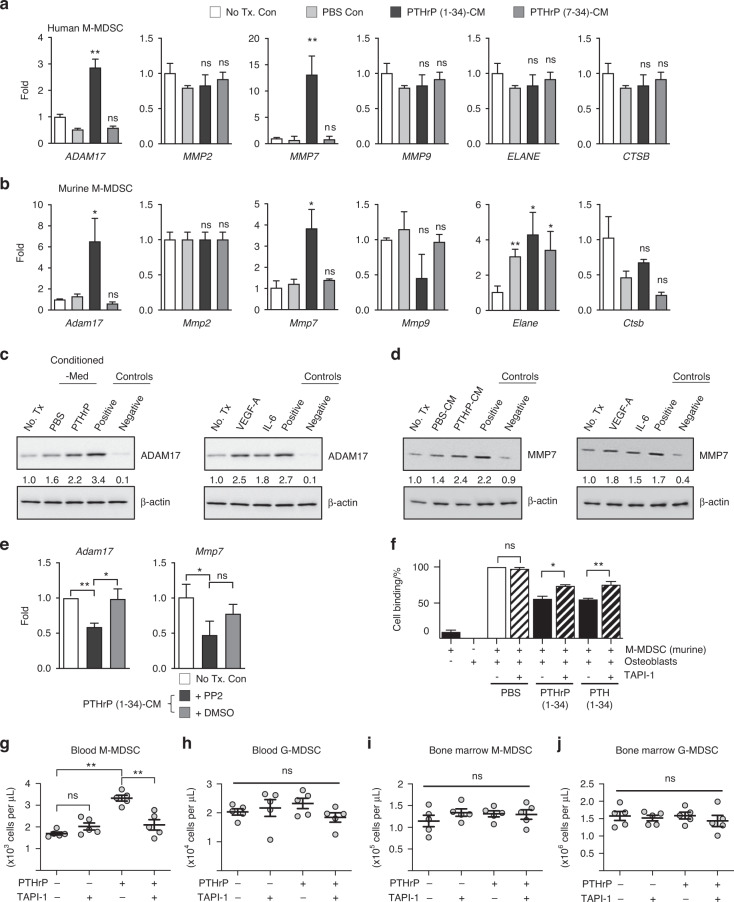
PTHrP-induced M-MDSC mobilization is dependent on A Disintegrin And Metalloproteinase (ADAM) 17 and MMP7. **a, b** RT‒qPCR results of human or murine M-MDSCs stimulated for 2 h with PBS- or PTHrP-conditioned media from osteoblasts. No treatment (No Tx) or PBS treatment was used as a control. **c, d** Immunoblotting analysis showing that ADAM17 (**c**) and MMP7 (**d**) expression in murine M-MDSCs was increased by PTHrP-conditioned media compared with no treatment (No Tx) or PBS controls. Recombinant VEGF-A or IL6 also increased ADAM17 and MMP7 expression. Cell lysates of NIH3T3 or murine PBMCs were used as positive and negative controls for ADAM17 expression, respectively. Cell lysates from A549 and PC-3 cell lines were used as positive and negative controls for MMP7 expression, respectively. β-Actin was used as a loading control. Band intensities normalized to no treatment controls are indicated. **e** RT‒qPCR results showing that *Adam17* and *Mmp7* expression in murine M-MDSCs was suppressed by PP2, an SFK-specific inhibitor. DMSO was used as a vehicle control. **f** In vitro cell binding assay with TAPI-1, an inhibitor of ADAM17 and MMPs. TAPI-1 treatment (50 μmol·L^−1^) reversed PTHrP(1-34)- or PTH(1-34)-induced MDSC release from osteoblasts. **(g-j)** TAPI-1 suppressed PTHrP(1-34)-induced M-MDSC mobilization in vivo. Female Balb/C mice were injected with rhPTHrP (1-34; 80 μg·kg^−1^) ± TAPI-1 (2 mg·kg^−1^, I.P.), followed by flow cytometric quantification of M- and G-MDSCs in blood and bone marrow. Dots represent individual samples, and horizontal lines represent the mean ± SEM. **P < 0.01; ns nonsignificant; Mann‒Whitney *U* test. Bar graph data (**a, b, e** and **f**) are the mean ± SEM (*n* = 3 per group). n.s. nonsignificant; *P < 0.05; **P < 0.01. One-way ANOVA with multiple group comparisons

We subsequently sought to determine the downstream mediators of pY419 SFK in M-MDSCs contributing to the disruption of M-MDSC and osteoblast binding. Human or murine M-MDSCs were isolated and stimulated for two hours with PBS- or PTHrP-conditioned media from human or murine osteoblasts, respectively, followed by reverse transcription-quantitative polymerase chain reaction (RT‒qPCR) for candidate protease genes, including *ADAM17*, *MMP2*, *7*, *9*, *ELANE* and *CTSB* (cathepsin B). Interestingly, *ADAM17* and *MMP7* were significantly increased in both murine and human M-MDSCs by PTHrP-conditioned media compared with no-treatment or PBS-conditioned media controls (Fig. 8a, b). In addition, VEGF-A and IL6 treatment increased ADAM17 and MMP7 protein expression in M-MDSCs (Fig. 8c, d). SFK suppression by PP2 decreased ADAM17 and MMP7 expression (Fig. 8e). These data collectively demonstrate that osteoblastic cytokines such as VEGF-A and IL6 induced by PTH1R activation result in ADAM17 and MMP7 expression in M-MDSCs via pY419 SFK. We subsequently performed a functional assay to test whether ADAM17 and MMP7 inhibition suppresses PTH/PTHrP-induced osteoblast-M-MDSC binding release. Figure 8f–j demonstrates that TAPI-1, an inhibitor of ADAM17 and MMPs, reversed PTHrP(1-34)- or PTH(1-34)-induced MDSC release from osteoblasts in vitro and the PTHrP(1-34)-induced M-MDSCs increase in the circulation of mice.

## Discussion

**Fig. 9:**
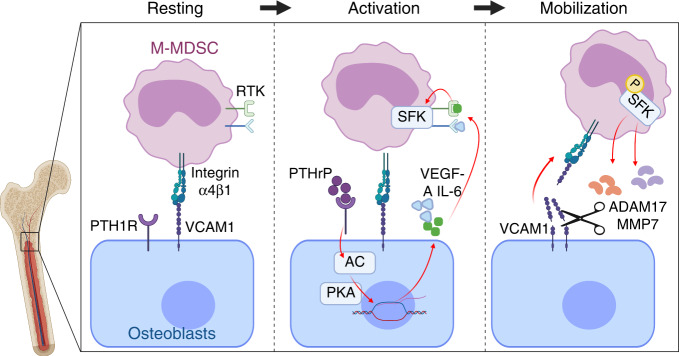
Schematic illustration of M-MDSC mobilization by PTH1R activation. M-MDSCs reside in the endosteal surface via expression of α4β1 integrin (VLA4) and osteoblastic expression of VCAM1. Tumor-derived PTHrP triggers the PTH1R-adenylate cyclase (AC)-Protein Kinase A (PKA) signaling axis in osteoblasts, and in turn, osteoblasts express VEGF-A and IL6, leading to activating phosphorylation of SFK in M-MDSCs. M-MDSCs subsequently express ADAM17 and MMP7 proteases, followed by shedding VCAM1 and mobilization of M-MDSCs into the circulation. RTK receptor tyrosine kinase. P phosphorylation. Created with Biorender.com

The present study demonstrated the molecular mechanism underlying how tumor cells exploit the skeletal system to augment MDSCs, which are important protumorigenic immune cells in the tumor microenvironment. As illustrated in Fig. 9, tumor-derived PTHrP circulates and triggers PTH1R on osteoblasts, leading to the expression of VEGF-A and IL6. As a result, M-MDSCs become activated via phosphorylation of SFK and express proteases such as MMP7 and ADAM17 to disengage the VLA4-VCAM1 axis-dependent tethering between M-MDSCs and osteoblasts. Mobilized M-MDSCs subsequently become available to the tumor tissue via circulation, contributing to escape from antitumoral immunity and tumor progression. The skeletal system is an essential partner for tumor progression because diverse subsets of stromal cells comprising the tumor microenvironment, such as immune cells, endothelial progenitor cells, tumor-associated macrophages, and tumor-associated fibroblasts, commonly originate in the bone marrow. Thus, stromal cells in the bone marrow, such as osteoblasts, are speculated to play active roles in supplying bone marrow-derived cells to the tumor microenvironment, but the precise mechanism remains to be elucidated. The present study focused on how the monocytic subset of MDSCs is mobilized from the bone marrow by tumor-derived factors. The majority of studies on MDSC development are centered on the recruitment of circulating MDSCs to tumor tissue or on the mechanism of immunosuppressive activities, and only a fraction of studies have investigated MDSC mobilization from the bone marrow into the circulation. As briefly summarized in the above introduction section, many cytokines and tumor-derived factors, mostly involved in HSC mobilization and/or myelopoiesis, potentially contribute to MDSC mobilization. However, the majority of studies are only observational, lack precise molecular mechanisms and are often confused with terms such as mobilization *vs*. expansion.^13,16,26,27^ In the present study, we employed in vivo mouse models and in vitro cell-binding assays using human patient MDSCs to show the molecular mechanisms of M-MDSC tethering and release to/from osteoblasts.

The applicability of our findings is multifaceted. Therapeutic strategies targeting MDSCs are currently under extensive investigation, and MDSC inhibition will significantly enhance the efficacy of immune checkpoint inhibitors. Numerous lines of evidence support that the number and function of MDSCs positively correlate with cancer progression as well as the reduced efficacy of immune checkpoint inhibitors.^28^ Currently, therapeutic targets to suppress MDSCs include pathways involved in MDSC expansion (e.g., STAT3 or prostaglandin E2 inhibitors), activation (e.g., inhibitors of interferon-γ, IL6, IL-1β, TNFα, *etc*.), metabolic pathways of MDSCs (e.g., hypoxia-inducible factor 1α, lactate accumulation, fatty acid oxidation, *etc*.), MDSC infiltration (e.g., Toll-like receptor 9, STAT3, VEGF-A, *etc*.), and MDSC depletion (e.g., cytotoxic chemotherapeutic agents and tyrosine kinase inhibitors such as nilotinib, dasatinib, sorafenib and ibrutinib) (refer to a review article^29^ written by Dr. Larry Kwak for more details). In contrast, none of the currently targeted pathways aims to shut down the MDSC supply from storage, the most fundamental step of MDSC development. The data in the present study support that multiple approaches using anti-PTHrP antibodies, protease inhibitors specific for ADAM17 or MMP7, and tyrosine kinase inhibitors suppressing SFK and/or VEGFR potentially block M-MDSC production in the bone marrow and consequently enhance the therapeutic efficacy of immune checkpoint inhibitors. PTHrP is a well-known factor for malignancy-induced hypercalcemia and tumor-bone interactions in breast cancer bone metastasis^30,31^; thus, anti-PTHrP antibodies have long been used to suppress cancer progression and metastasis.^32–35^ However, anti-PTHrP antibodies have never been tested in combination with immune checkpoint inhibitors in cancer patients or murine tumor models, which is an important direction for further studies. Likewise, dasatinib, a selective SFK inhibitor, was shown to significantly reduce PTHrP-induced M-MDSC mobilization in vivo (Fig. 7c), supporting that SFK tyrosine kinase inhibitors could be repositioned to suppress MDSCs and to enhance cancer immunotherapy. Previously, dasatinib was shown to reduce tumor-infiltrating myeloid cells.^36^ In addition, Sun et al. demonstrated that inhibition of SFK by dasatinib reduced MDSCs in a head and neck cancer mouse model and that among nine family members of SFK, Lyn is an important therapeutic target in MDSCs.^37^ Indeed, the same group subsequently showed that combinatorial treatment with dasatinib and anti-CTLA4 antibody reduced tumor growth and MDSC numbers in a murine tumor model.^38^ Data in our present study not only logically extended and confirmed the previous findings but also provided the precise molecular mechanism of how SFK inhibitors could synergistically cooperate with immune checkpoint blockade.

Our proposed molecular mechanism of MDSC mobilization, however, raises many more important questions. First, while MDSCs are considered universally important stromal cells in the tumor microenvironment, PTHrP is not expressed by every tumor type. Thus, we reason that additional tumor-derived factors or mechanisms mediating tumor-bone interactions and MDSC mobilization warrant extensive further investigation. Second, given that recombinant human PTH (teriparatide, Forteo ®) is currently used as a bone anabolic agent for osteoporotic patients and that PTH and PTHrP share the common receptor PTH1R, increased PTH levels in the circulation due to rhPTH treatment or hyperparathyroidism will potentially increase MDSCs in the blood and reduce T-cell immunity. Questions such as whether hyperparathyroidism patients or those who receive rhPTH have an increased number and/or activity of MDSCs will be worth addressing in large clinical cohorts. If the increased level of PTH is found to be associated with increased MDSCs, the patients who receive rhPTH should be thoroughly screened for cancer because MDSCs can fuel the growth of occult or microscopic tumor cells. Third, the roles of proteolytic cleavage and shedding of VCAM1 and/or VLA-4 in the mobilization of MDSCs and other types of hematopoietic lineage cells need further investigation. The proposed mechanism in this paper is supported by assays using multiple inhibitors, including TAPI-1, an ADAM17 inhibitor. Garton et al. showed ADAM17-dependent shedding of VCAM1 and measurement of soluble VCAM1 in the culture supernatant.^39^ Lévesque et al. further demonstrated that proteolytic cleavage of VCAM1 expressed by bone marrow stromal cells is an essential step of hematopoietic progenitor cell mobilization in response to G-CSF^40^ and that hematopoietic mobilizing agents such as G-CSF and cyclophosphamide increase proteases such as neutrophil elastase and cathepsin G, transforming the bone marrow into a highly proteolytic environment.^41^ Our study added further details on the VCAM1 proteolytic cleavage-dependent regulation of M-MDSCs in the bone marrow of tumor hosts. In contrast, further evidence, such as detection of cleaved VCAM1 in the bone marrow flush or in the circulation, will strengthen the interpretation. In conclusion, the data in this study explain how M-MDSCs, essential bone marrow-derived cells in the TME, are regulated in the bones of cancer patients and provide a scientific basis for novel therapeutic strategies suppressing M-MDSCs and boosting immune checkpoint inhibitors.

## Materials and methods

### Flow cytometric analysis and sorting

Murine blood samples were collected from the retro-orbital sinus, and bone marrow cells were collected by flushing hindlimb long bones. Erythrocytes were lysed by ACK lysis buffer, followed by staining with anti-mouse monoclonal antibodies including CD16/CD32 (Mouse Fc Block™), CD45 (30-F11), CD11b (M1/70), Gr-1 (RB6-8C5), Ly-6C (AL-21), Ly-6G (1A8), CCR2 (SA203G11), CD3 (17A2), CD4 (RM4-5), CD8 (53-6.7), CD49b (DX5) and B220 (RA3-6B2). BioGems™ Viability Dye 506 or 780 was used to exclude dead cells. Flow cytometric analysis was performed using BD LSRFortessa™ X-20 or FACSCanto™ II cytometers and FlowJo™ software version 10.6., and flow cytometric sorting was performed using a BD FACSAria™ III sorter and FACSDIVA™ software version 8.0. Gating strategies are presented in Fig. 1b and Fig. S1.

### Human blood samples

Peripheral blood mononuclear cells (PBMCs) of female breast cancer patients were obtained from an ongoing clinical study. The study was approved by the institutional review board of the Korea University Medical Center Anam Hospital (No. ED17183). Briefly, whole blood samples were collected from female breast cancer patients who visited the outpatient clinic of the Korea University Anam Hospital, Seoul, Korea, from October 2017 to October 2019. PBMCs were isolated by Ficoll-Paque™ density-gradient centrifugation, followed by washing and resuspension in FACS buffer (phosphate-buffered saline (PBS) with 2% fetal bovine serum (FBS) and 2 mmol·L^−1^ EDTA). Cells were stained with anti-human monoclonal antibodies, including CD45 (HI30), CD11b (ICFR44), CD14 (MφP9) and CCR2 (K036C2), followed by flow cytometric sorting (BD FACSAria™ II). All patients signed written informed consent before enrollment, and the study was conducted in accordance with the International Conference on Harmonization Good Clinical Practice guidelines and the provisions of the Declaration of Helsinki.

### Cells

Murine primary calvarial osteoblasts were isolated as previously described.^42–44^ Briefly, the calvariae of 2~5-day-old C57BL6/J mouse pups were dissected and subjected to serial digestion with alpha modification minimal essential media (αMEM) with type 1 collagenase (125 units per mL) and 1× penicillin/streptomycin antibiotics for 15 min each. After the first and second fractions were discarded, the third and fourth fractions were plated and expanded in complete αMEM, followed by cryopreservation in CELLBANKER® cell freezing media (Amsbio). Cells were thawed and used without passaging more than once. Thermolabile SV40 large T antigen-transformed hFOB 1.19 human fetal osteoblasts cells (ATCC) were cultured in Dulbecco’s modified Eagle’s media (DMEM)/F-12 media at restrictive temperature for experiments and were used without passaging more than three times. HUVECs (ATCC) were cultured and expanded in Endothelial Cell Growth Medium 2 (produced by and purchased from PromoCell). NIH3T3 fibroblasts were cultured and expanded in complete DMEM. For murine primary osteoclasts, monocytes were isolated from the bone marrow flush of tumor-naïve Balb/C mice by gradient centrifugation, followed by treatment with αMEM with M-CSF (30 ng·mL^−1^) and receptor activator of nuclear factor kappa B ligand (RANKL, 50 ng·mL^−1^) for four days. *PTH1R* knockdown osteoblasts were established by transducing primary calvarial osteoblasts or hFOB1.19 cells with *PTH1R*-targeting shRNA lentiviral particles (Santa Cruz Biotechnology SC-36327-V). Scrambled-sequence shRNA lentiviral particles were used as controls. VCAM1-overexpressing MCF7 cells were generated by transfecting wild-type MCF7 cells with a VCAM1 overexpression vector (Origene RC209761). Cells were regularly confirmed to be free of mycoplasma by PCR tests and authenticated for matching short tandem repeat DNA profiles of the original cell lines.

### Mouse models

All animal experiments were approved by the Institutional Animal Care and Use Committee of the Korea University Medical Center. Mouse tumor models were established as previously described.^19,44^ Briefly, luciferase- and tdTomato-labeled 4T1 murine breast cancer cells (gifted by Dr. Eun Kyoung Choi, Asan Medical Center, Seoul, Korea) were injected intratibially (1 × 10^4^ cells per 20 µL) or subcutaneously (5 × 10^4^ cells per 100 µL) into 7-week-old female Balb/C mice. Osteocalcin (*Ocn*)-*Cre*:*ROSA*^*mT/mG*^ reporter mice were generated by crossing mT/mG mice (*Gt(ROSA)26Sor* ^*tm4(ACTB-tdTomato,-EGFP)Luo*^/J, The Jackson Laboratory Strain No. 007576) with *Ocn*-*Cre* mice (B6N.FVB-Tg(BGLAP-cre)1Clem/J, The Jackson Laboratory Strain No. 019509). B16F10 murine melanoma cells were injected subcutaneously into 7-week-old C57BL6 mice or *Ocn*-*Cre*:*ROSA*^*mT/mG*^ mice. For continuous PTHrP stimulation, Alzet® osmotic pumps releasing 80 μg·kg^−1^ per day recombinant human (rh) PTHrP (amino acids 1–34) were surgically transplanted into the subcutaneous space of 7-week-old male mice.^19^ For analysis of inhibitors of M-MDSC mobilization, mice were intraperitoneally administered anti-IL6 antibody (10 mg·kg^−1^), anti-VEGF-A antibody (5 mg·kg^−1^), TAPI-1 (2 mg·kg^−1^), and/or dasatinib (15 mg·kg^−1^, P.O) prior to 30 min of rhPTHrP (1–34. 80 μg·kg^−1^) subcutaneous administration.

### T-cell suppression assay

Murine CD3^+^ T cells were isolated from the spleens of tumor-naïve C57BL6 or Balb/C mice using T Cell Enrichment Columns (R&D Systems, MTCC-25) and stained with carboxyfluorescein succinimidyl ester (CFSE, 1 μmol·L^−1^). Murine M-MDSCs were isolated from the bone marrow of B16F10 tumor-bearing C57BL6 and 4T1 tumor-bearing Balb/c mice or Alzet osmotic pump-implanted Balb/c mice using a Mouse MDSC Isolation Kit (Miltenyi Biotec 130-094-538). CD3^+^ T-cell purity after column enrichment was confirmed to be higher than 75% (Fig. S1c). T cells and MDSCs were cocultured in the presence of IL-2 (10 IU per mL) and Dynabeads® CD3/CD28 T-cell activators (bead:T-cell ratios = 0.5:1 or 0.2:1) for three days, followed by flow cytometric quantification of proliferating T cells.

### Isolation of M-MDSCs

Human or murine M-MDSCs were isolated by flow cytometric cell sorting (BD FACSAria II) from the PBMCs of human breast cancer patients or the spleens or tibiae of tumor-bearing mice.

### In vitro cell binding assay

An in vitro osteoblast-MDSC binding assay was performed by modifying a previously published hematopoietic stem cell-osteoblast binding assay.^45,46^ Briefly, human or murine osteoblasts (2.5 × 10^3^ or 1 × 10^4^, respectively) were seeded on 96-well flat-bottom black-wall polystyrene plates and incubated overnight. Human or murine M-MDSCs were sorted by flow cytometry and stained with CFSE. Subsequently, M-MDSCs were added to the osteoblast monolayer culture and incubated for 30 min in a 5% CO_2_ 37 °C incubator with experimental treatments (e.g., PTH or PTHrP). Unbound cells were washed with PBS, and fluorescence intensity (excitation 485 nm, emission 515 nm) was measured with a plate reader. The fluorescence intensity of the PBS-treated control group was considered 100% binding, and a reduction in fluorescence intensity was interpreted as reduced cell binding (%).

### Fluorescence and confocal microscopy

For DiD cell membrane staining, adherent cells (osteoblasts and MCF7 cells) were trypsinized and resuspended in serum-free complete media (10^6^ cells in 1 mL of media), followed by the addition of Vibrant^TM^ DiD Cell-Labeling Solution (5 μL) and incubation for 20 min at 37 °C. Cells were plated on confocal dishes. The next day, MDSCs were sorted by flow cytometry and stained with CFSE. If needed, nuclear counterstaining was performed using 4’,6-diamidino-2-phenylindole (DAPI) or Hoechst 33342. Fluorescence or confocal microscopic images were captured using an EVOS FL AUTO2 (Thermo Fisher) or confocal microscope (LSM800 or LSM900, Zeiss), respectively. For in vivo histological images, hindlimbs from B16F10 tumor-bearing C57BL6 mice or *Ocn*-*Cre*:*ROSA*^*mT/mG*^ reporter mice were dissected and fixed with 4% (w/v) paraformaldehyde for 3 days, followed by decalcification in 14% EDTA for 3 days and 30% sucrose in PBS for 1 day. For cryosectioning, tissues were then embedded in Tissue-Tek® OCT compound, and 5 μm thick sections were cut and mounted onto microscopic slides. For immunohistochemistry, sections were stained with anti-CD11b (M1/70), anti-Ly6C (ER-MP20), anti-alkaline phosphatase (ALP, Abcam Catalog No. 224335, polyclonal) and anti-VCAM1 (Genetax Catalog No. GTX12133, polyclonal) antibodies.

### Electron microscopy

M-MDSCs isolated from 4T1 tumor-bearing mice were cocultured with adherent murine calvarial osteoblasts on a confocal dish, fixed with 2.5% glutaraldehyde solution overnight at 4 °C and washed in PBS. Fluorescence images were captured by a widefield microscope (EVOS M7000, Thermo Fisher). Subsequently, the specimen was postfixed with 1% osmium tetroxide. After washing with distilled water, the specimen was gradually dehydrated with increasing concentrations of ethanol series (50%, 70%, 90%, and 100%) and hexamethyldisilazane (HMDS) series (3:1, 1:1 and 1:3 ethanol:HMDS, and 100% HMDS). After drying in air, the specimen was mounted on SEM stubs followed by platinum coating at 15 mA for 60 s using a sputter coater (E-1045, Hitachi). Surface images were observed using SEM (TeneoVS, FEI) in SE mode at 10 kV and 0.1 nA with an ETD detector at the same location where fluorescence images were taken.

### Statistical analysis

Statistical analysis was performed using GraphPad Prism™ version 8.0. All data were tested for normality by the Shapiro‒Wilk test, and Student’s *t* test (for normally distributed samples) or the Mann–Whitney *U* test (for nonparametric analysis) was used to compare groups. One-way ANOVA with multiple group comparisons analysis was performed to compare normally distributed multiple groups. All statistical analyses were two-sided.

## Supplementary information

Supplemental Figure 1

Supplemental Figure 2

Supplemental Figure 3

Supplemental Figure 4

Supplemental Movie 1

Supplemental Figure Legends
