# Supplementary figures and images for: Mobilization of monocytic myeloid-derived suppressor cells is regulated by PTH1R activation in bone marrow stromal cells
Source: Bone Res. 2023 Apr 21;11:22. doi: 10.1038/s41413-023-00255-y (PMC10121701; doi:10.1038/s41413-023-00255-y)

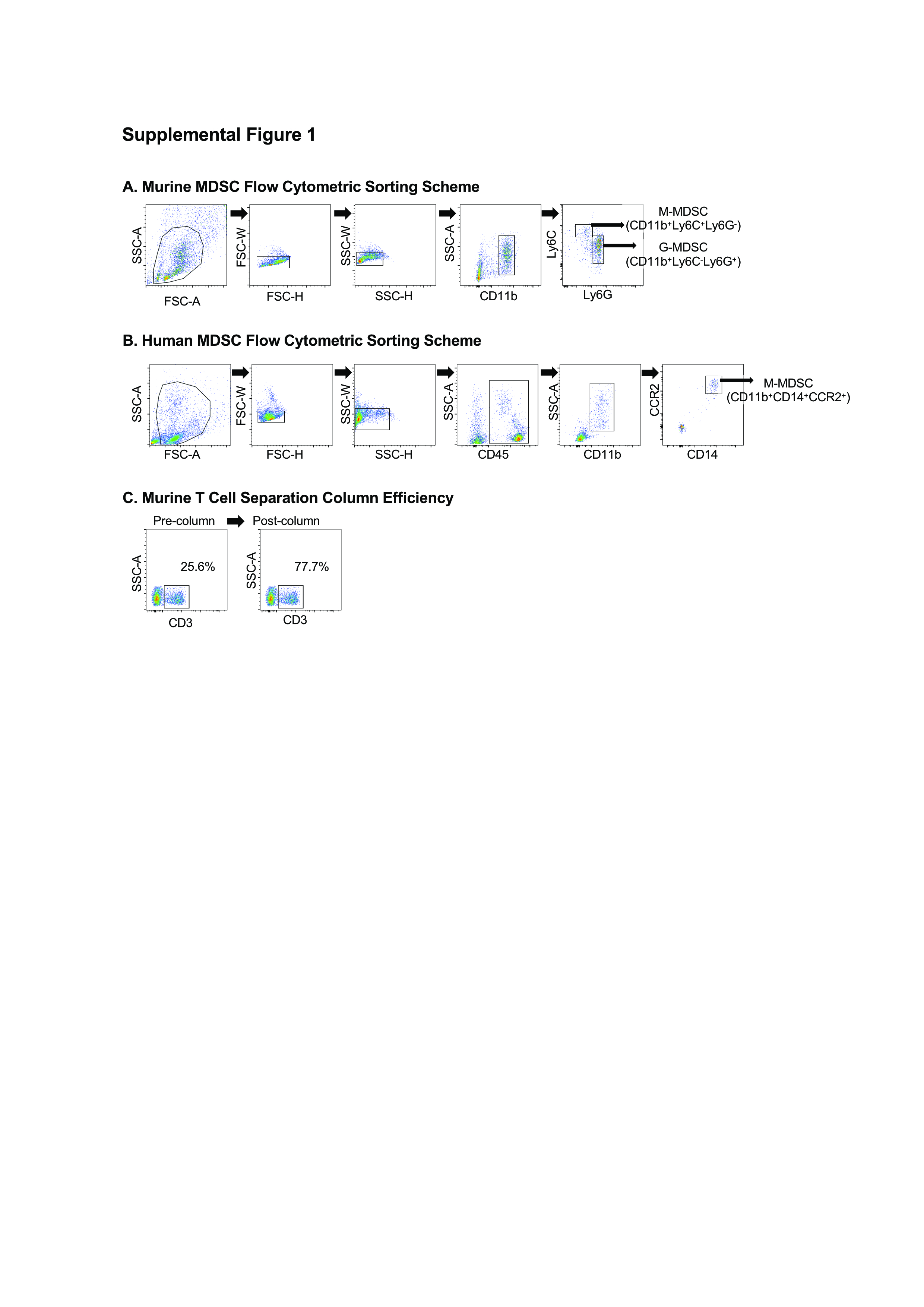

Supplement: Supplementary file 1 — Supplemental Figure 1 [file 41413_2023_255_MOESM1_ESM.jpg]

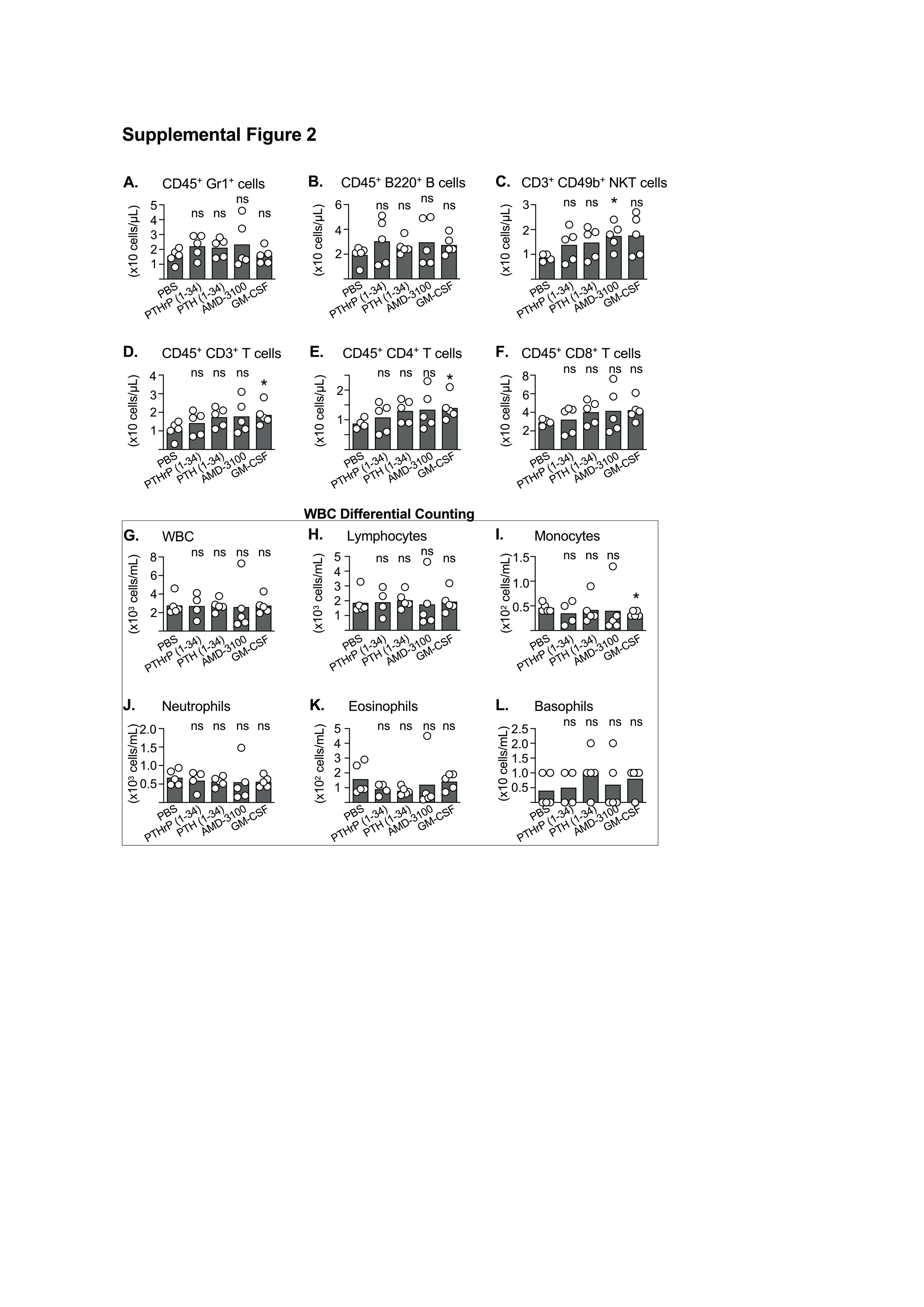

Supplement: Supplementary file 2 — Supplemental Figure 2 [file 41413_2023_255_MOESM2_ESM.jpg]

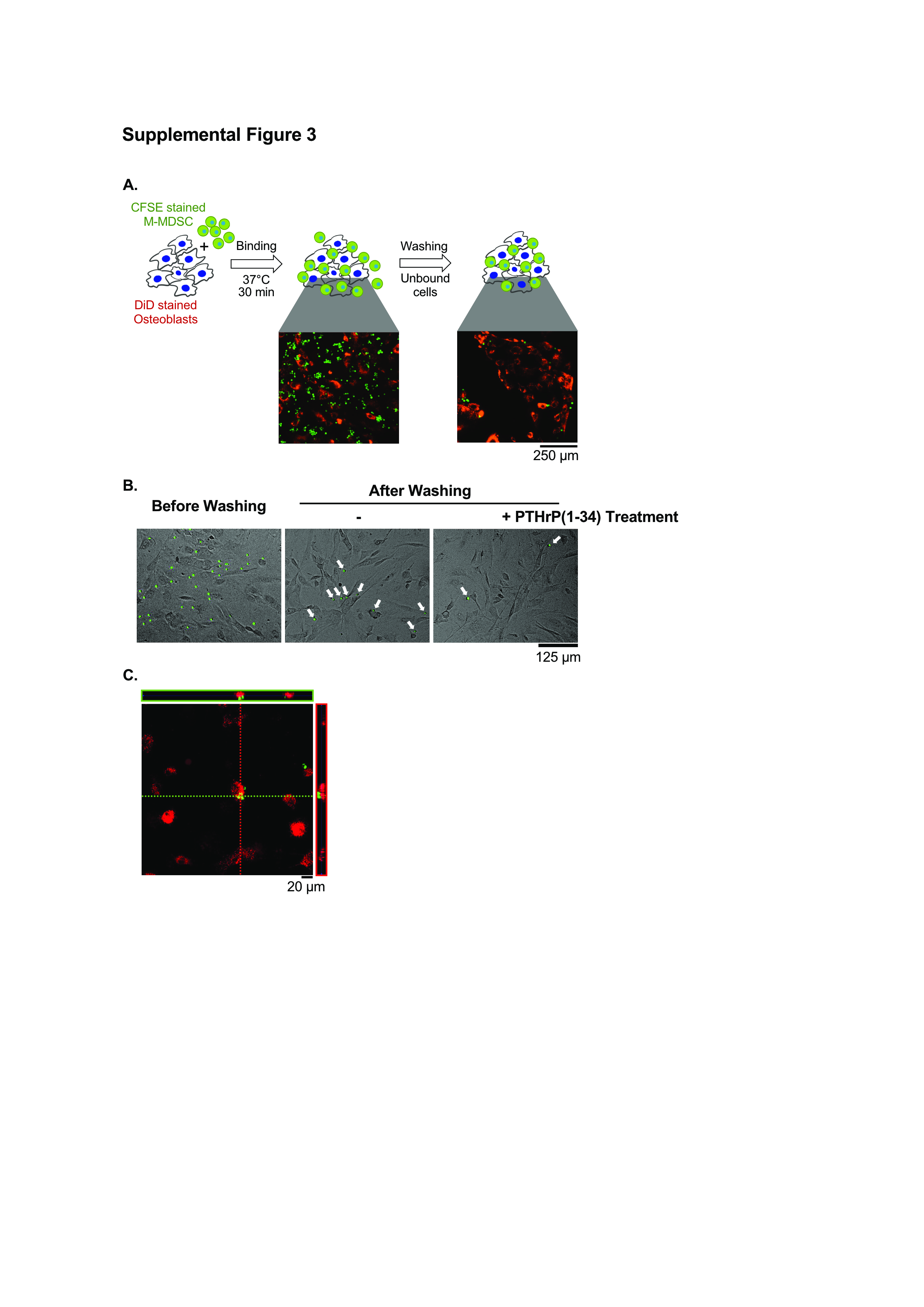

Supplement: Supplementary file 3 — Supplemental Figure 3 [file 41413_2023_255_MOESM3_ESM.jpg]

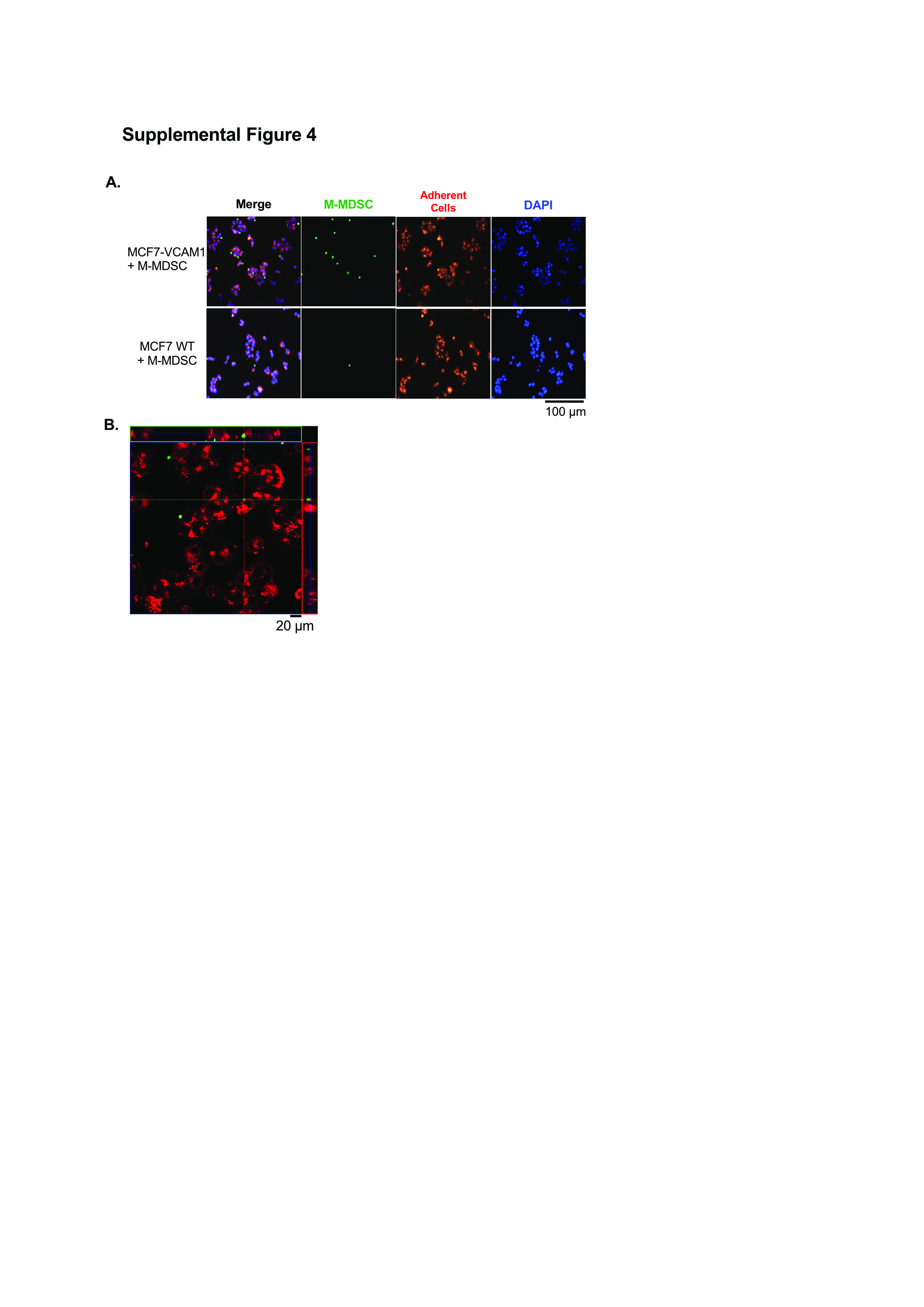

Supplement: Supplementary file 4 — Supplemental Figure 4 [file 41413_2023_255_MOESM4_ESM.jpg]
