# Supplementary material for: Mobilization of monocytic myeloid-derived suppressor cells is regulated by PTH1R activation in bone marrow stromal cells
Source: Bone Res. 2023 Apr 21;11:22. doi: 10.1038/s41413-023-00255-y (PMC10121701; doi:10.1038/s41413-023-00255-y)
Supplement: Supplementary file 6 — Supplemental Figure Legends [file 41413_2023_255_MOESM6_ESM.docx]

**SUPPLEMENTAL FIGURE LEGENDS**

**SUPPLEMENTAL FIGURE 1 Flow cytometry gating strategies and T-cell separation column purity**

**(A and B)** Flow cytometry gating strategies for murine (A) and human (B) MDSCs.

**(C)** Cell purity of murine splenic CD3^+^ T cells was confirmed by flow cytometry. Briefly, murine CD3^+^ T cells were isolated by pulverizing the spleens of tumor-naïve C57BL6 mice and subsequently by T-Cell Enrichment Columns. T-cell purity after column enrichment was confirmed to be higher than 75%.

**SUPPLEMENTAL FIGURE 2 The effects of PTH(1-34), PTHrP(1-34), AMD-3100 and GM-CSF on subsets of hematopoietic lineage cells**

Female Balb/C mice received a single injection of PBS, rhPTHrP (1-34; 80 µg/kg), rhPTH (1-34; 80 µg/kg), AMD3100 (5 mg/kg) and GM-CSF (50 µg/kg). Blood samples were collected and analyzed by multicolor flow cytometry (**A-F**) or WBC differential counting (**G-L**).

**SUPPLEMENTAL FIGURE 3. *In vitro* cell binding assays for osteoblast and MDSC interactions**

**(A)** Schematic representation of the *in vitro* cell binding assay. Vivrant® DiD-labeled murine calvarial osteoblasts were cultured on a confocal dish (1×10^5^ cells, 3.14 cm^2^) and incubated overnight. Murine M-MDSCs were isolated by flow cytometric sorting from 4T1 tumor-bearing mouse tibias, followed by CFSE labeling and coculture with DiD-labeled osteoblasts for 30 minutes (M-MDSCs 1×10^5^ cells). Subsequently, unbound cells were washed with PBS. Microscopic images of the pre- and postwashing steps are shown.

**(B)** Fluorescence microscopic images of CFSE-stained M-MDSCs (isolated from 4T1 tumor-bearing mice; 1×10^4^ cells) overlaid with phase-contrast microscopic images of unstained murine calvarial osteoblasts (1×10^4^ cells/well, seeded on a clear-bottom and black-sidewall 96-well plate) before and after PBS washing steps ± PTHrP (1-34, 10 nM).

**(C)** Orthogonal views from different planes (x-y, x-z or y-z) of confocal microscopy showing murine primary calvarial osteoblasts (Vibrant® DiD-NIR dye; orange) and murine M-MDSCs (CFSE; green). Refer to Supplemental Movie 1 for a 3D view of osteoblast and M-MDSC binding.

**SUPPLEMENT FIGURE 4. Ectopic expression of VCAM1 in MCF7 cells**

**(A)** Fluorescence microscopic images show murine M-MDSC binding to VCAM1-overexpressing but not to parental MCF7 cells. Orange, Vibrant® DiD-NIR dye (MCF7). Blue, DAPI. Green, CFSE (M-MDSC).

**(B)** Orthogonal views from different planes (x-y, x-z or y-z) of confocal microscopy showing MCF7-VCAM1 (Vibrant® DiD-NIR dye; red) and murine M-MDSCs (CFSE; green).

**SUPPLEMENTAL Movie 1. *In vitro* cell binding assays for osteoblast and MDSC interactions**Z-stack serial sections of Vibrant® DiD-NIR dye-stained (orange) murine calvarial osteoblasts and CFSE-stained (green) murine M-MDSCs were captured using a Zeiss LSM-900 confocal microscope, followed by deconvolution, 3D reconstruction and movie file rendering using the Huygens Professional (SVI) image processing software package.
